# Corilagin inhibits angiotensin II-induced atrial fibrosis and fibrillation in mice through the PI3K-Akt pathway

**DOI:** 10.22038/IJBMS.2024.73281.15928

**Published:** 2024

**Authors:** Xiaogang Zhang, Bei Tian, Xinpeng Cong, Zhongping Ning

**Affiliations:** 1 Cardiovascular Department, Shanghai University of Medicine and Health Sciences Affiliated Zhoupu Hospital, Shanghai, China

**Keywords:** Akt, Angiotensin II, Atrial fibrillation, Atrial fibrosis, Corilagin, PI3K

## Abstract

**Objective(s):**

Corilagin (Cor) is reported as beiing hepatoprotective, anti-inflammatory, antibacterial, and anti-oxidant, while the effect on atrial fibrosis remains unknown. Therefore, we investigated the protective effect of Cor in angiotensin II (Ang II)-induced atrial fibrosis and atrial fibrillation (AF).

**Materials and Methods:**

C57BL/6 mice (male, 8–10 weeks, n = 40) were subcutaneously infused either with saline or Ang II (2.0 mg/kg/day) and Cor (30 mg/kg) intraperitoneally injected 2 hr before Ang II infusion for 4 weeks. Mice were grouped into the control group (n=8), Cor group (n=8), Ang II group (n=8), and Ang II + Cor group (n=8). Morphological, histological, and biochemical examinations were performed.* In vivo*, transesophageal burst pacing was used to generate AF.

**Results:**

Cor treatment markedly reduced Ang II-induced AF development in mice. Ang II + Cor therapy potentially decreased the atrial fibrotic area. It significantly decreased the increase in smooth muscle alpha-actin (α-SMA), CTGF, Collagen I, and Collagen III expressions brought on by Ang II treatment. Moreover, Ang II + Cor treatment remarkably decreased the malondialdehyde (MDA) content, whereas superoxide dismutase (SOD) and catalase (CAT) activities were potentially increased (all, *P*<0.001). In addition, Ang II + Cor significantly reduced Ang II-induced interleukin 1 beta (IL-1β), interleukin 6 (IL-6), and tumor necrosis factor-alpha (TNF-α) concentrations in atrial tissues. Furthermore, Cor significantly inhibited Ang II-induced p-PI3K, p-Akt, and NF-κB p-p65 protein expression in atrial tissues.

**Conclusion:**

Our data speculated that Cor could have a protective effect against Ang II-induced atrial fibrosis and AF via down-regulation of the PI3K-Akt pathway.

## Introduction

Atrial fibrillation (AF) is a frequent and dangerous arrhythmia that can cause thromboembolic incidents and stroke. Fibrous tissue accumulates in the atria, the heart’s upper chambers, as a result of the disease known as atrial fibrosis. The structural and electrical remodeling of the atria that results in atrial fibrosis can be brought on by several conditions, including age, cardiac failure, valvular disease, or ischemia (1). Congestive heart failure (CHF) in humans and animal models of pacing-induced heart failure exhibit atrial interstitial fibrosis (2, 3). Therefore, exploring the detailed molecular mechanisms underlying AF is urgently needed to provide new targets to develop therapeutic procedures for AF treatment.

Atrial fibrosis in AF can be accelerated by angiotensin II (Ang II) (4). Transforming Growth Factor-β1 (TGF-β1) is released by platelets and interacts with fibroblasts to cause Ang II-induced AF (5). The Ang II type 1 receptor-TGF-β1/Smad signaling pathway causes myocardial fibrosis due to AF (6). By stimulating the ERK1/2 and phosphoinositide 3-kinase (PI3K)/Akt signaling pathways in vascular smooth muscle cells (VSMCs), Ang II controls a variety of physiological and pathological responses (7). In addition, the PI3K/Akt pathway is activated by Ang II, which increases the proliferation of natural killer cells and T-cell lymphoma cells (8). However, the PI3K-Akt pathway regulates several cellular functions, including proliferation, survival, inflammation, and growth (9). A transcription factor known as NF-kB regulates the expression of genes related to immunological and inflammatory responses (10). Moreover, several previous studies reported that the PI3K-Akt-NF-kB pathway might have a role in controlling the development and progression of AF and fibrosis by influencing cell survival, apoptosis, growth, contractility, and gene expression (11, 12). Therefore, the role of the PI3K-Akt signaling pathway in Ang II-induced atrial fibrosis and AF in mice is still unclear.

**Figure 1:**
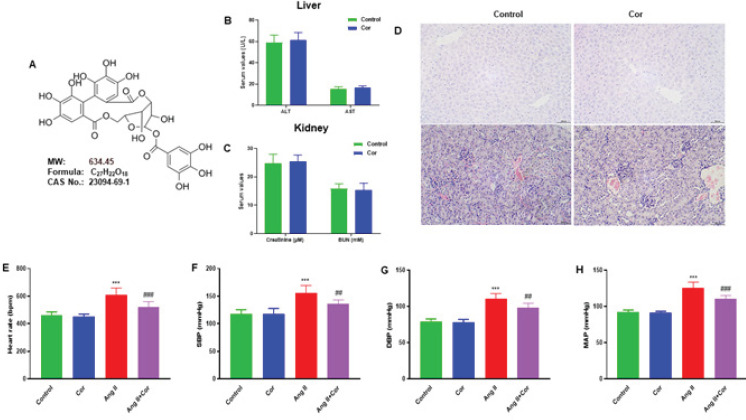
Safety and physiological functions of corilagin in mice

Corilagin (Cor) is an ellagitannin from the tannin family and is a main bioactive component in many medicinal plants (13). The chemical structure of Cor is shown in Figure 1A. Previous studies reported Cor’s biological and pharmaceutical effects are diverse, including hepatoprotective, anti-inflammatory, antibacterial, anti-oxidant, anti-hypertensive, anti-diabetic, and anti-tumor characteristics (14). It can promote apoptosis and prevent cell proliferation, inhibiting the formation of several cancer cells (13). Cor has been shown to have hepatoprotective properties in treating several liver-related conditions, including hepatic cancer, drug-induced liver damage, and liver fibrosis (15). Recent studies demonstrated that by controlling the IL-13, GATA3, and miR21/smad7/extracellular regulated protein kinase (ERK) signaling pathways, Cor could reduce hepatic fibrosis caused by schistosomiasis (16-19). However, the potential impact of Cor on angiotensin II-induced atrial fibrosis and fibrillation via the PI3K-Akt pathway remains unknown. The present study investigated Cor’s protective effects against Ang II-induced atrial fibrosis and AF in mice and explored the underlying mechanisms precisely.

## Materials and Methods

***Animals and treatment***

C57BL/6 mice (male, 8–10 weeks, n = 40) were used to establish the AF model. Mice were maintained in the SPF laboratory animal room under environmentally controlled conditions (22 ± 2 °C; humidity 40%) with a 12 hr light/dark cycle. Mice were subcutaneously infused with Ang II (2.0 mg/kg/day) (20) or the same volume saline using osmotic mini-pumps for 4 weeks. The Cor (30 mg/kg/day, HY-N0462, MedChemExpress) or the same volume of saline was administered intraperitoneally once a day for 4 weeks. The first injection of Cor was 2 hr before the beginning of Ang II infusion. The mouse systolic blood pressure (SBP) and diastolic blood pressure (DBP) were measured by a tail-cuff system (Softron BP98A; Softron Tokyo, Japan) on day 28 before the electrocardiogram (ECG). As required by the Guide for the Care and Use of Laboratory Animals (NIH Publication No. 85-23, revised 1996), this study received approval from the Animal Care and Utilize Committee of Shanghai Pudong Zhoupu Hospital, China.

***Arrhythmia inducibility and duration***

After 4 weeks of Cor injection and Ang II infusion, the mice were anesthetized (intraperitoneal injection of 1% pentobarbital sodium). An 8-electrode catheter (Japan Lifeline, Tokyo, Japan) was inserted via the jugular vein and advanced into the right atrium and ventricle. An automated stimulator produced a 2-sec burst to induce AF. ECG was recorded with a computer-based data acquisition system (MadLab-4C/501H, ZS Dichuang Co., Ltd., Beijing, China). The parameters were: voltage: 20 V; current: 4 mA; wave width: 6 ms. AF was defined as a rapid, irregular atrial rhythm with periodic R-R intervals of 1 sec. The duration of AF was defined as the time interval from the end of burst pacing to the first P-wave (21).

***Animal grouping and drug administration***

After the successful pacing procedure, all 32 alive mice were equally assigned as follows: (a) The Control group (n=8) was intraperitoneally injected and infused with saline. (b) The Cor group (n=8) was intraperitoneally injected with v (30 mg/kg/day) and infused with saline. (c) The Ang II group (n=8) was intraperitoneally injected with saline and infused with Ang II (2.0 mg/kg/day). (d) The Ang II + Cor group (n=8) was intraperitoneally injected with Cor (30 mg/kg/day) and infused with Ang II (2.0 mg/kg/day). Eight (8) dead mice were discarded from all experiments. The administration of Cor and Ang II lasted for 28 consecutive days.

***Sample collection***

Serum and atrial tissue were obtained after intraperitoneal injection of 1% pentobarbital sodium to anesthetize the mice. For RT-qPCR and western blot, some tissue was immediately frozen in liquid nitrogen; for histological investigation, another tissue was preserved in 4% paraformaldehyde.

***Biochemical analysis***

Serum was collected to measure ALT (C009-2-1, Nanjing Jiancheng Bioengineering Institute, Nanjing, China), AST (C010-2-1, Nanjing Jiancheng), Creatinine (ab65340), and BUN (ab83362) levels using commercial kits. The atrial tissues were homogenized (10%, w/v) for measuring MDA (S0131S, Beyotime, Shanghai, China) content, SOD (S0109, Beyotime), and CAT (S0051, Beyotime) activities using commercially available kits.

***Histology of atrium***

The degrees of liver and kidney injury were evaluated by Hematoxylin/eosin (HE) staining. Slides were stained with Masson’s trichrome to evaluate the extent of atrial fibrosis. The fibrosis extent was evaluated by the ratio of the area of fibrotic to normal myocardium and was reported as collagen volume fraction.

***Immunohistochemistry***

IHC, or immunohistochemistry, is a technique that uses specific antibodies to stain the samples to identify the distribution and location of target antigens in cells or tissues. Atrial tissue was embedded in paraffin and then cut into 4 μm serial sections after the tissue had been treated with 4% paraformaldehyde. Masson’s trichrome was used to color the slides, or they were treated with rabbit polyclonal antibodies against α-SMA antibody (1:400; ab5694; Abcam, UK) for 20 min at 37 °C before being exposed to a biotinylated HRP conjugated secondary antibody. The procedure was carried out by the developer›s instructions. The slides were examined using an optical microscope (IX51, Olympus, Japan).

***Immunofluorescence***

The Immunofluorescence procedure was carried out as previously explained (22). The atrial tissue of mice was obtained from frozen sections. TUNEL (C1086, Beyotime), and DHE (S0063, Beyotime) were applied after three PBS washes. The cells were then counterstained with DAPI and examined under an inverted microscope (IX51, Olympus, Japan). TUNEL and DHE were carried out following the manufacturer’s recommendations.

***Oxidative stress***

To obtain cell lysate, the atrial tissue was homogenized. The cell lysate was then stored at -80 °C in the refrigerator. A commercial kit was used to measure MDA (S0131S, Beyotime, Shanghai, China), SOD (S0109, Beyotime) and CAT (S0051, Beyotime) activities, and a previously published protocol was followed to estimate the amount of oxidative stress (23).

***Enzyme-linked immunosorbent assay (ELISA)***

The concentrations of IL-1β (MLB00C, R&D Systems), IL-6 (M6000B, R&D Systems), and TNF-α (MTA00B, R&D Systems) in serum samples were determined using ELISA kits. The 450 nm absorbance was measured in a microplate reader, and the cytokine concentrations were calculated using the standard curve.

***Reverse transcription-quantitative polymerase chain reaction (RT-qPCR)***

Total RNA was extracted from atrial tissue using TRizol (Invitrogen, USA). Total RNA was reverse-transcribed into complementary DNA (cDNA). SYBR Green reagent (TaKaRa, Japan) was used in an ABI Prism 7700 Real-Time PCR equipment (Applied Biosystems, USA) to amplify the mRNA by Real-Time Quantitative PCR (RT-qPCR). The thermal cycler program was as follows: samples were heated to 95 °C for 3 min to denature them, then underwent 39 cycles of amplification and quantification (95 °C for 15 sec and 58 °C for 15 sec, respectively), followed by a final extension cycle (72 °C for 90 sec). Using the 2-ΔΔCt formula, the relative gene expression was normalized to the internal control, GADPH (24). The list of primers utilized in this investigation is presented in Table 1.

***Western blotting***

The protein was extracted with RIPA buffer, and after centrifuging the lysates to produce the supernatants, the protein concentrations could be measured using a BCA protein assay kit. Fifty micrograms of protein samples were loaded for 10% SDS-PAGE electrophoresis before being transferred to a PVDF membrane (Millipore, Bedford, MA). A TBS solution containing 5% skim milk blocked the membrane. The membrane was incubated with the following p-PI3K (p85, Tyr458) (1:400, ab278545, rabbit monoclonal, Abcam), t-PI3K ( 1:500, ab302958, rabbit monoclonal, Abcam), p-Akt (Ser473) (1:400, ab81283, rabbit monoclonal, Abcam), t-Akt (1:400, ab8805, rabbit polyclonal, Abcam), p-p65 (Ser536) (1:400, #3033, rabbit monoclonal, Cell Signaling), t-p65 (1:400, #8242, rabbit monoclonal, Cell Signaling), and GAPDH (1:1000, ab9485, rabbit polyclonal, Abcam) antibodies overnight at 4 °C. Using HRP-conjugated secondary antibodies (1:2000), the immunological reactivity of these target proteins was identified. The protein band was observed using ECL (Thermo, Waltham, MA, USA), and band density was measured using image processing software (Bio-Rad, Hercules, CA, USA).

***Statistical analysis***

All data were presented as the mean + standard deviation (SD) and repeated at least three times. Statistical analyses were performed using SPSS 20.0 (SPSS, Chicago, IL, USA). Student’s t-test was used to analyze the differences between the two groups’ tests. One-way ANOVA and the *post hoc* Tukey test analyzed the differences between multiple groups. AF incidence in the five groups was compared using Fisher’s exact test. Statistical significance was set as *P*<0.05.

## Results

***Preliminary evaluation of the safety and physiological characteristics in mice***

Mice were intraperitoneally injected with Cor (30 mg/kg/day) to examine the possible effects on safety and physiological characteristics in mice following Cor administration. The *in vivo* systemic toxicity of Cor (30 mg/kg/day) was assessed after 28 days of intraperitoneal administration. The results showed no adverse effects on the mice’s liver and renal function indicators, including ALT, AST, Creatinine, and BUN (Figure 1B, 1C). On the liver and kidney slices from the saline- and Cor-infused mice, hematoxylin and eosin (H&E) stained results revealed the same consistent impact (Figure 1D). Mice were administered Ang II (2.0 mg/kg/day) and Cor (30 mg/kg/day) intraperitoneally for 28 days to assess physiological characteristics. The results showed that Cor reduced the heart rate, systolic blood pressure (SBP), diastolic blood pressure (DBP), and mean arterial pressure (MAP) of Ang II-infused mice (Figure 1E-H). 

***Corilagin decreased Ang II-induced AF***

**Figure 2:**
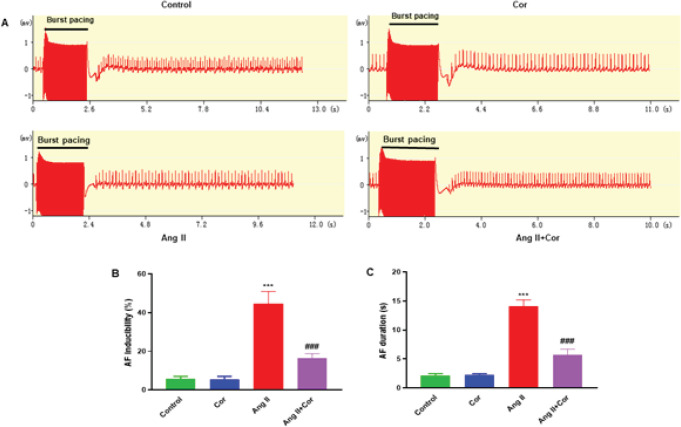
Corilagin decreases atrial fibrillation (AF) inducibility and AF duration induced by burst pacing

 Mice were infused with Ang II with or without Cor (30 mg/kg/day) for 28 days to examine the function of Cor in controlling the development of AF. Transesophageal rapid atrial pacing was done first, and the ECG was recorded. Typical AF assaults were observed in the mice in the AF group (Figure 2A). The inducibility and persistence of AF were investigated in Ang II-infused mice injected with or without Cor. The inducibility of AF was dramatically increased after administering Ang II treatment compared with the control group. In mice treated with Ang II +, Cor significantly reduced the inducibility of AF caused by Ang II, whereas Cor alone had no effect (Figure 2B). The overall length of AF was significantly reduced in the Ang II + Cor treated mice compared to the Ang II infusion, with no significant change observed after the Cor therapy alone (*P*>0.001) (Figure 2C). The results showed that Cor potentially inhibits Ang II-induced AF development in mice.

***Corilagin reduced Ang II-induced atrial fibrosis***

**Figure 3:**
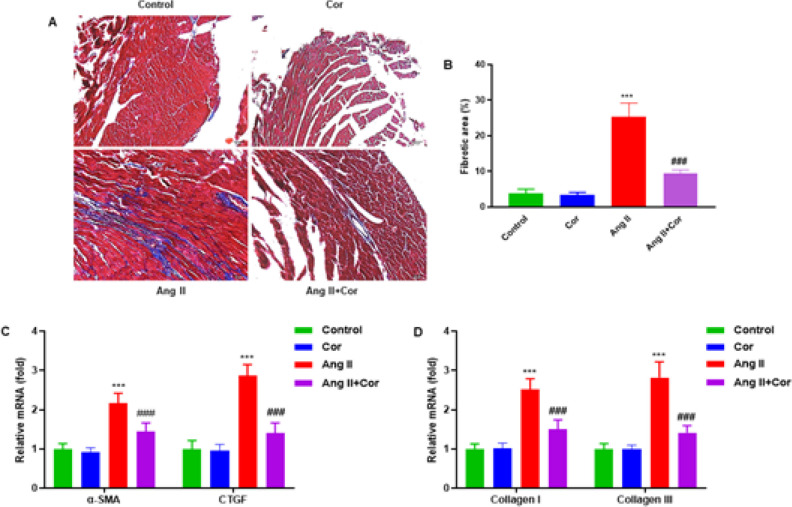
Corilagin suppresses Ang II-induced atrial fibrosis

The effect of Cor on atrial fibrosis, a hallmark attribute of atrial remodeling, was next examined. Ang II treatment significantly increased the atrial fibrotic area compared to the control group. The Ang II + Cor treatment dramatically decreased the increased atrial fibrotic area caused by Ang II, whereas only the Cor treatment had no impact observed (Figure 3A, 3B). In addition, RT-qPCR was performed to measure the mRNA expression of fibrotic-related genes, including α-SMA, CTGF, Collagen I, and Collagen III. The results showed that Ang II + Cor treatment significantly decreased the increase of α-SMA, CTGF, Collagen I, and Collagen III expressions caused by Ang II, whereas no effect was observed in the Cor group (Figure 3C, 3D).

***Corilagin suppressed oxidative stress in Ang II-induced atrial fibrosis***

**Figure 4:**
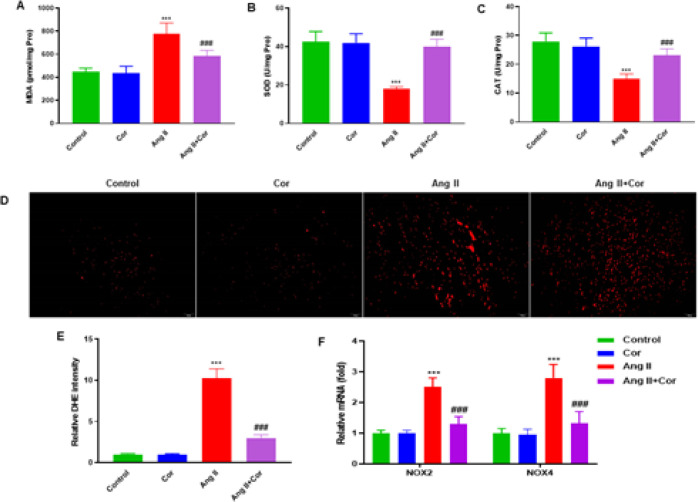
Corilagin suppresses oxidative stress in the atrial tissue of mice with Ang II infusion

Several important oxidative stress markers were measured in the atrial tissue lysate, including malondialdehyde (MDA), superoxide dismutase (SOD), and catalase (CAT). The MDA content was decreased in the Ang II + Cor group compared to the Ang II group (*P*<0.001) in Figure 4A. On the other hand, SOD and CAT activities were significantly increased in the Ang II + Cor group compared to the Ang II group (*P*<0.001 for SOD and *P*<0.001 for CAT, respectively) (Figure 4B, 4C). DHE staining revealed that Ang II treatment significantly increased the generation of ROS in the atrial tissue. After pretreatment with Ang II + Cor, the DHE-positive cells were also significantly decreased (*P*<0.001) compared to the Ang II group (Figure 4D, 4E). Additionally, RT-qPCR was used to assess NOX2 and NOX4 mRNA expression. Compared to the Ang II group, the results demonstrated that the expression of NOX2 and NOX4 was considerably decreased by the Ang II + Cor treatment (*P*<0.001 for NOX2 and *P*<0.001 for NOX4, respectively) (Figure 4F).

***Corilagin attenuated Ang II-induced inflammation of atrial tissues***

**Figure 5:**
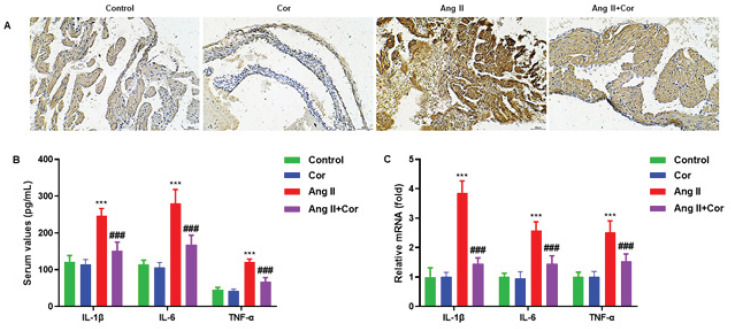
Corilagin inhibits Ang II-induced inflammation of atrial tissues

In mice atrial tissues, the effects of Cor on inflammation were examined. A study using immunohistochemistry revealed that Cor dramatically reduced the number of cells with high-intensity IL-1β cells in the atrial tissue of mice (Figure 5A). ELISA was performed to measure IL-1β, IL-6, and TNF-α concentrations in the serum of mice. The results showed that Ang II + Cor significantly reduced Ang II-induced IL-1β, IL-6, and TNF-α concentrations in atrial tissues (Figure 5B). In addition, RT-qPCR was carried out to determine the mRNA expression of IL-1β, IL-6, and TNF-α in atrial tissues. We observed the consistent reduction of IL-1β, IL-6, and TNF-α mRNA levels in mice’s atrial tissues (Figure 5C). 

***Corilagin suppressed Ang II-induced p-PI3K, p-Akt, and NF-*** **κB** ***p-p65 protein expression in atrial tissues***

**Figure 6:**
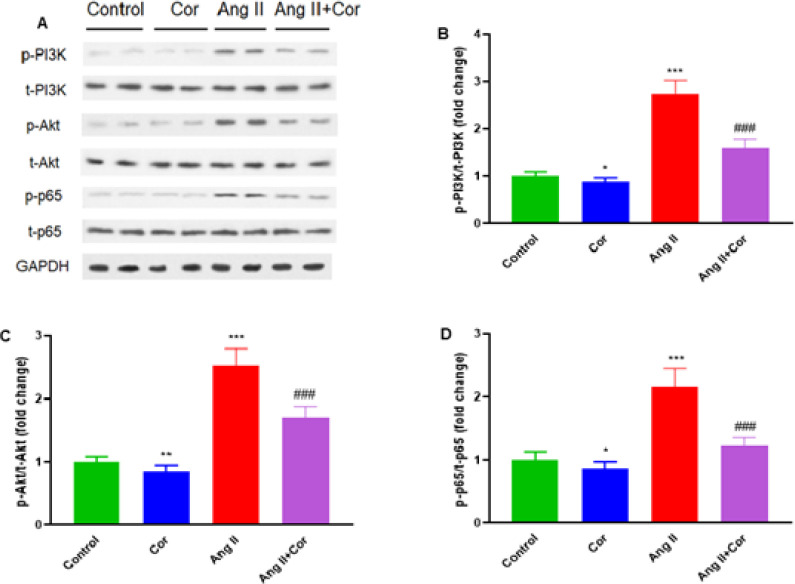
Corilagin modulated PI3K-Akt-NF-κB signaling in atrial tissue of Ang II-treated mice

Western blot analysis was used to determine the protein levels of key components of the PI3K-Akt-NF-kB signaling pathway in Ang II-induced mice (Figure 6A). Ang II + Cor treatment potentially attenuated the protein expression of p-PI3K, p-Akt, and NF-**κB** p-p65, compared to the Ang II group (all, *P*<0.001) (Figures 6B, 6C, and 6D). The results demonstrated that Cor significantly inhibited Ang II-induced p-PI3K, p-Akt, and NF-**κB** p-p65 protein expression in atrial tissues.

(A) Structural formula, molecular weight, chemical formula, and CAS number of corilagin. Corilagin was intraperitoneally injected (30 mg/kg/day) into mice for 28 days to evaluate its systematic toxicity *in vivo*. (B) Hepatic and (C) renal function indicators of mice were measured, including ALT, AST, Creatinine, and BUN. (D) Hematoxylin and eosin (H&E) stained hepatic and renal slices from the saline and corilagin-injected mice—scale bar: 50 μm. Mice were infused with Ang II (2.0 mg/kg/day) and intraperitoneally injected with corilagin (30 mg/kg/day) for 28 days. Changes in (E) heart rate, (F) systolic blood pressure (SBP), (G) diastolic blood pressure (DBP), and (H) mean arterial pressure (MAP) were monitored. Data are presented as mean ± SD (n=8 in each group).

***P<0.001 vs Control group; ##*P*<0.01, ###*P*<0.001 vs Ang II group

**Table 1:** List of primer oligonucleotide sequences used in this study in mice

**Genes**	**Forward primer (** **5′-3′)**	**Reverse primer (** **5′-3′)**
α-SMA	TCCTGACGCTGAAGTATCCGATA	GGCCACACGAAGCTCGTTAT
CTGF	CCAGACCCAACTATGATGCG	GTGTCCGGATGCACTTTTTG
Collagen I	GCTCCTCTTAGGGGCCACT	CCACGTCTCACCATTGGGG
Collagen III	TCCCCTGGAATCTGTGAATC	TGAGTCGAATTGGGGAGAAT
NOX2	TGGCGATCTCAGCAAAAGGT	ACCTTGGGGCACTTGACAAA
NOX4	ACCAAATGTTGGGCGATTGTG	GATGAGGCTGCAGTTGAGGT
IL-1β	GAAATGCCACCTTTTGACAGTG	TGGATGCTCTCATCAGGACAG
IL-6	AACCACGGCCTTCCCTACT	CATTTCCACGATTTCCCAGA
TNF-α	CGTCGTAGCAAACCACCAA	GGGCAGCCTTGTCCCTTGA
GAPDH	ACTCCACTCACGGCAAATTC	TCTCCATGGTGGTGAAGACA

α-SMA: α-smooth muscle actin; CTGF: Connective tissue growth factor; NOX2: NADPH Oxidase 2; IL-1β: Interleukin-1β; IL-6: Interleukin-6; TNF-α: Tumor necrosis factor-α; GAPDH: Glyceraldehyde-3-phosphate dehydrogenase

(A) Representative atrial electrogram recordings. Solid underlines indicate burst pacing. (B) Incidence of successful AF in mice of each group. (C) The average duration of AF induced by burst pacing. Data are presented as mean ± SD (n=8 in each group)

***P<0.001 vs Control group; ###*P*<0.001 vs Ang II group

(A) Atrial tissues were stained with Masson trichrome, and representative images were shown. (B) The extent of atrial fibrosis was quantified by calculating the percentage of fibrotic area. RT-qPCR was performed to measure the mRNA expression of fibrotic-related genes, including (C) α-SMA and CTGF and (D) Collagen I and Collagen III. Data are presented as mean ± SD (n = 8 in each group).

***P<0.001 vs control group; ###*P*<0.001 vs Ang II group

The atrial lysate was analyzed for oxidative stress indicators, and the levels of (A) MDA, (B) SOD and (C) CAT were quantified. (D) Representative images of DHE staining of atrial tissues. (E) Quantification of DHE intensity. (F) RT-qPCR was carried out to determine the mRNA expression of NOX2 and NOX4. GAPDH serves as an internal control. Data are presented as mean ± SD (n = 8 in each group)

***P<0.001 vs control group; ###*P*<0.001 vs Ang II group.

MDA: Malondialdehyde; SOD: Superoxide dismutase; CAT: Catalase; DHE: Dihydroethidium

(A) Representative images of IL-1β by immunohistochemistry in atrial tissue of mice (Magnification, 200×). (B) ELISA was performed to measure IL-1β, IL-6, and TNF-α concentration in the serum of mice. (C) RT-qPCR was used to determine the mRNA expression of IL-1β, IL-6, and TNF-α in atrial tissue. Data are presented as mean ± SD. n = 5 mice per group in triplicate. ***P<0.001 vs control group; ###*P*<0.001 vs Ang II group

(A) Western blot shows the representative bands of p-PI3K, p-Akt, and NF-κB p-p65 in mouse atria. Quantification of immunoblotting results are shown, including (B) p-PI3K, (C) p-Akt, and (D) NF-κB p-p65. Protein expression was normalized to their total proteins, respectively. Data are presented as mean ± SD (n = 8 in each group).

***P<0.001 vs. control group; ###*P*<0.001 vs. Ang II group

## Discussion

The current study aimed to examine Cor’s possible impact on Ang II-induced atrial fibrosis and AF. The results showed that Cor’s had no adverse effects on the hepatic and renal tissues and no significant alterations in the mice stimulated with Ang II regarding body weight, heart rate, or systolic blood pressure (SBP). Cor treatment potentially inhibited Ang II-induced AF, atrial fibrosis, and oxidative stress and attenuated Ang II-induced inflammation of atrial tissues. In addition, Cor administration significantly inhibited Ang II-induced p-PI3K, p-Akt, and NF-κB p-p65 protein expression in atrial tissues. Therefore, our results speculate that Cor could be potentially protected against Ang II-induced atrial fibrosis and AF.

AF is an arrhythmia that can have catastrophic consequences. Several factors contribute to the onset and continuation of AF disorders (25). Atrial fibrosis is the most prevalent cause of structural remodeling in AF patients and the structural basis of AF persistence. Remodeling of the left atrium (LA), including structural, electrical, and autonomic remodeling, is associated with AF (27, 28). Atrial fibrosis development in the LA explicitly assists in forming, developing, and sustaining AF (28). Myocardial fibrosis is a frequent characteristic in both experimental and human AF (29). The current investigation showed that Cor treatment inhibited Ang II-induced atrial fibrosis, retained atrial conduction uniformity, and reduced AF susceptibility in mice.

Angiotensin II is the primary regulator of cardiac oxidative stress, increasing the generation of reactive oxygen species (ROS) in the cardiovascular system by activating membrane-bound NOX, endoplasmic reticulum stress, and mitochondrial oxidative stress (30). ROS can potentially lead to myocardial fibrosis, promoting the appearance and development of AF. This produces a potentially deadly positive feedback loop, leading patients to AF and, as a result, increased fibrosis (31). Previous research found that targeting ox-CaMKII loss in oxidation-resistant CaMKII MMVV mice was enough to inhibit the proarrhythmic response of Ang II in AF (32). CaMKII, they reasoned, is an upstream signal of ROS that can be triggered by NOX (33). However, in our study, NOX2 and NOX4 expression was increased in Ang II-induced mice, leading to mitochondrial damage, exacerbating oxidative stress and fibrosis, and, ultimately, vulnerability to AF. The Ang II + Cor treatment significantly reduced the expression of NOX2 and NOX4.

The present study showed that Ang II + Cor administration significantly inhibited the Ang II-induced inflammation of atrial tissues. Ang II is an increased risk factor for AF and may lead to atrial fibrosis and inflammation, increasing AF inducibility (34). Inflammation plays a role in the pathogenesis of AF, and excessive inflammatory mediators transfer into atrial tissue, affecting its structural and electrical characteristics (35). However, recent research has shown that Cor significantly reduces inflammation by reducing the production of proinflammatory cytokines (36), which is consistent with our current study findings.

The proteins phosphoinositide 3-kinase (PI3K), protein kinase B (AKT), and nuclear factor-kappa B (NF-kB) are involved in a variety of cellular processes, including differentiation, survival, and inflammation (37). Recent studies reported that by inhibiting the NF-**κB** and PI3K/AKT signaling pathways, Cor could prevent bone loss and reduce osteoclastogenesis (38). In addition, Ang II also triggers these pathways and is crucial in renal disorders and podocyte damage (37). In the present study, we observed that p-PI3K, p-Akt, and NF-B p-p65 protein expression potentially increased in Ang II-induced mice, whereas Ang II + Cor treatment significantly inhibited. Therefore, Cor could be protected against Ang II-induced atrial fibrosis and AF in mice by down-regulating the PI3K-Akt pathway. 

***Limitations***

There are several drawbacks to this present study. No dose-dependent effect was investigated in Cor’s effectiveness. The underlying findings of the study have not been verified in preclinical or clinical settings. Additional research is required to determine the causative mechanism underlying Cor’s protective impacts against Ang II-induced atrial fibrosis and AF. However, our study provides significant aspects of Cor’s protective effects against atrial fibrosis and AF.

## Conclusion

Our research investigated the possible protective effect of Cor on Ang II-induced atrial fibrosis and AF on mice models. Our growing evidence shows that Cor therapy significantly reduced Ang II-induced atrial fibrosis and AF by inhibiting the PI3K-Akt signaling pathway. It will be necessary to verify these results in clinical and preclinical settings in the future to provide additional confirmation.

## Authors’ Contributions

X Z conceived the study, provided methodology and visualization, and wrote the original draft. B T and X C contributed to investigation, software analysis, data curation, and validation. Z N helped with project administration, supervision, manuscript revision, funding acquisition, and resources.

## Conflicts of Interest

The authors declare no conflicts of interest with other people or organizations.
